# Long non-coding RNA XIST predicts worse prognosis in digestive system tumors: a systemic review and meta-analysis

**DOI:** 10.1042/BSR20180169

**Published:** 2018-06-21

**Authors:** Xuefang Liu, Xinliang Ming, Wei Jing, Ping Luo, Nandi Li, Man Zhu, Mingxia Yu, Chunzi Liang, Jiancheng Tu

**Affiliations:** 1 Department of Laboratory Medicine, Clinical Laboratory Medicine and Center for Gene Diagnosis, Zhongnan Hospital of Wuhan University, Wuhan 430071, China; 2 Department of Clinical Laboratory, The First Affiliated Hospital of Zhengzhou University, Key Laboratory of Laboratory Medicine of Henan, Zhengzhou 450000, China

**Keywords:** clinicopathological characteristic, LncRNA, prognosis, XIST

## Abstract

Increasing studies are indicating that long non-coding RNA (lncRNA) X-inactive specific transcript (XIST) is associated with the prognosis of cancer patients. However, the results have been disputed. Therefore, we aimed to further explore the prognostic value and clinical significance of XIST in various types of cancers. Then, we focussed our research on the comparison of the predictive value of XIST between digestive system tumors and non-digestive system tumors. We performed a systematic search by looking up PubMed, Embase, Cochrane Library, Web of Science, and Medline (up to 3 January 2018). Fifteen studies which matched our inclusion criteria with a total of 920 patients for overall survival and 867 patients for clinicopathological characteristics were included in this meta-analysis. Pooled hazard ratios (HR) and odds ratios (ORs) with their corresponding 95% confidence intervals (95% CIs) were calculated to summarize the effects. Our results suggested that high expression levels of XIST were associated with unfavorable overall survival in cancer patients (pooled HR = 1.81, 95% CI: 1.45–2.26). Additionally, we found that XIST was more valuable in digestive system tumors (pooled HR = 2.24, 95% CI: 1.73–2.92) than in non-digestive system tumors (pooled HR = 1.22, 95% CI: 0.60–2.45). Furthermore, elevated expression levels of XIST were connected with distant metastasis and tumor stage. XIST was correlated with poor prognosis, which suggested that XIST might serve as a novel predictive biomarker for cancer patients, especially for patients of digestive system tumors.

## Introduction

Cancers have become a major cause of mortality for human health over the past decade [1]. Digestive system tumors, consisting mainly of gastric cancer, colorectal cancer, hepatocellular carcinoma, esophageal cancer, pancreatic cancer, and gallbladder cancer, have been counted as the main cause for all the cancer-related mortality worldwide [2]. Due to the lack of sensitive imaging methods and biomarkers, large number of cancer patients are mostly detected at advanced stage, and the 5-year survival rate still remains far from satisfactory [3]. Recently, researchers have been devoting themselves in identifying the novel tumor biomarkers associated with tumor screening, diagnosis, prognosis, and evaluation of therapeutic efficacy to improve their survival status [4,5]. As the incidence of cancers is on the rise [6], it is clinically urgent and necessary to explore reliable biomarkers which can predict prognosis in malignant cancers, especially for digestive system cancers.

Previous studies have shown that long non-coding RNAs (lncRNAs) with a length greater than 200 nts play a crucial role in cancer development, which can act as tumor suppressor genes or oncogenes [7]. The aberrant expression of lncRNAs has been confirmed in different kinds of cancers by comparing expression of tumor and paired adjacent non-cancerous tissue [8–10], which suggests that lncRNAs may be involved in the cancer occurrence and development.

The lncRNA X-inactive specific transcript (XIST) is necessary for X-chromosome inactivation in female mammals [11–13], which can recruit polycomb repressive complex 2 (PRC2), thus mediating repression of the entire chromosome and also maintaining the silent state. Recently, increasing studies have shown that XIST can function as a tumor suppressor gene or oncogene in different types of cancers, including nasopharyngeal carcinoma [14], non-small-cell lung cancer [15], esophageal squamous cell carcinoma [16], colorectal cancer [17], breast cancer [18], gastric cancer [19], cervical squamous cell carcinoma [20], pancreatic cancer [21], prostate cancer [22], bladder cancer [23], and so on. However, these studies have been moderately limited because of relatively small sample sizes and some results are controversial. Therefore, the present systematic review and quantitative meta-analysis were performed to assess the prognostic and clinicopathological roles of XIST in different types of cancers and further to evaluate its predictive value in digestive system tumors.

## Materials and methods

### Search strategy and literature selection

Up to 3 January 2018, eligible articles which evaluated XIST as a biomarker for the prognosis or clinicopathological characteristics for cancer patients were searched in several international databases, including PubMed, Embase, Web of Science, and Cochrane. To increase search sensitivity, we used a strategy involving both Medical Subject Heading terms and free-text words. The searched terms were listed as follows: (‘lncRNA-’, ‘XIST’) and (‘cancer’ or ‘carcinoma’ or ‘tumor’ or ‘neoplasm’) and (‘prognosis’ or ‘prognostic’ or ‘survival’ or ‘outcome’ or ‘metastasis’) or (‘characteristic’ or ‘clinical features’). With respect to the retrieved articles, we first excluded the ones not relevant to cancers. Then, titles, abstracts, and full texts of retrieved articles were carefully scanned to eliminate the ones without usable data.

### Inclusion and exclusion criteria

Inclusion criteria were as following: (i) articles evaluating the relationship between XIST expression level and overall survival or clinicopathological parameters of any type of cancer; (ii) articles for which we can obtain hazard ratio (HR) and 95% confidence interval (95% CI) directly or extract HR and 95% CI from survival curves indirectly; (iii) articles published in English; (iv) available full-text articles; (v) research on humans. Exclusion criteria were as following: (i) articles absence of overall survival; (ii) excluding earlier or smaller sample size ones for duplicate publications as well as duplicate data; (iii) reviews, letters, or laboratory studies lacking original data; (iv) articles published in languages other than English.

### Data extraction and quality assessment

Two investigators (Xuefang Liu and Xinliang Ming) extracted all the essential information from enrolled articles independently. According to the inclusion and exclusion criteria, we deliberately extracted the following information: (i) general information including primary author, year, age, and gender of the patients enrolled, detection method, normative reference, and sample size; (ii) clinicopathological characteristics including lymph node metastasis, distant metastasis, differentiation, and tumor stage; (iii) the relationship between expression level of XIST and overall survival; (iv) survival curves. Quality assessment was performed according to the reporting recommendations for tumor marker prognostic studies (REMARK) guideline.

### Statistical analysis

The meta-analysis was conducted with Stata SE12 Software (STATA Corp, College Station, Texas, U.S.A.). Odds ratios (ORs) with corresponding 95% CIs were performed to analyze the association of XIST expression level with lymph node metastasis, distant metastasis, differentiation, and tumor stage. HRs with corresponding 95% CIs were utilized to estimate the relationship between the expression level of XIST and prognosis of cancer patients. In detail, HRs were extracted by using two methods: (i) we directly obtained HRs from the publication; (ii) we estimated the HRs and 95% CIs by choosing several survival rates at specified times from the Kaplan–Meier survival curves using Engauge Digitizer version 4.1. Heterogeneity across the enrolled studies was quantitated with the *I^2^* statistics. The random-effect model was performed if heterogeneity was present (*I^2^* ≥ 50% or *P*≤0.05). Otherwise, the fixed-effect model was more appropriate. We evaluated publication bias by constructing a funnel plot with Begg’s test.

### Trial sequential analysis

The standards for meta-analysis with high quality should be as religious as single randomized trial [24]. It usually requires multiple tests in meta-analysis, thus increasing random error. Systematic or random errors might lead to unreliable results in meta-analyses. Trial sequential analysis (TSA) can create a line of TSA boundary value by adjusting threshold for significant level with sparse data. A *priori* information size (APIS) is considered as the sample size needed for a reliable and conclusive result. There appeared four cases: (i) the cumulative Z-curve crosses the traditional Z-value but fails to cross the TSA boundary value, indicating that more trials are necessary to confirm the positive result; (ii) the cumulative Z-curve crosses both of them, meaning that trials show positive results in advance; (iii) the cumulative Z-curve crosses neither of them, which suggests that that more trials are necessary to confirm the negative result; (iv) the cumulative Z-curve crosses APIS value but cannot cross the traditional Z-value. It can be assumed that there was no significance between the experimental group and the control group. It was performed by setting the required power and risk for type I and type II errors. In our study, TSA was performed by setting an overall type-I error of 5%, a 15% relative risk reduction (RRR), and a statistical test power of 80%.

## Results

### Studies included

**Figure 1:**
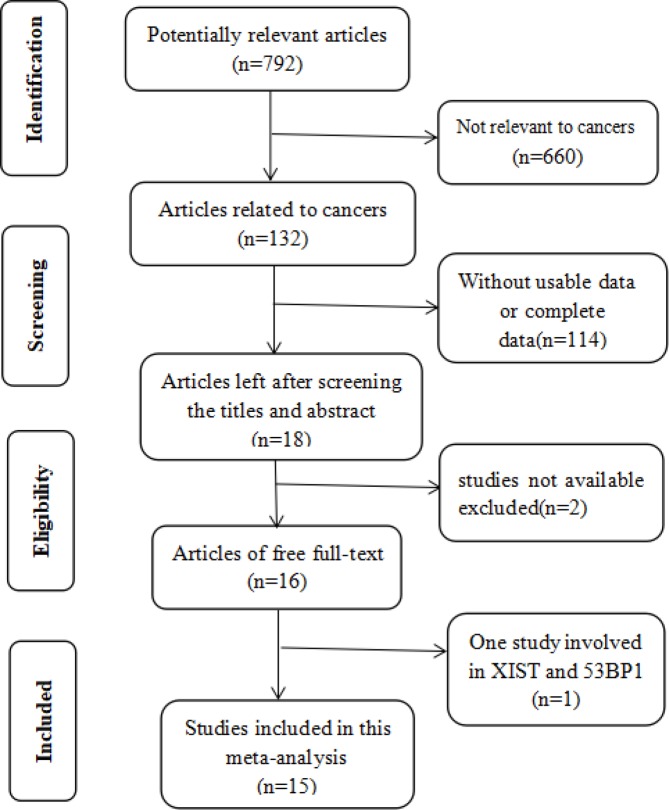
Flow diagram of the search and selection process

The literature search and selection process are shown in Figure 1. A total of 792 articles were retrieved from PubMed, Embase, Cochrane Library, Web of Science, and Medline, amongst which 132 studies were relevant to cancers. After a careful view of the title, abstract, key words, and the full texts, 114 articles did not include the usable data. Amongst the 18 articles left, two articles were not available. One article with survival curves made of XIST and 53BP1 was finally excluded. Subsequently, 15 articles were enrolled in the current meta-analysis, including 11 studies for prognosis and 12 for clinicopathological characteristics. The relevant information of the studies included for prognosis and clinicopathological characteristics were respectively shown in Tables 1 and 2.

**Table 1:** Characteristics of studies included for prognosis

Author	Year	Country	Type	Method	Reference	Case number (high/low)	Survival analysis	Analysis type	Follow-up months	HR availability	Quality score
Ma et al. [40]	2017	China	GC	RT-PCR	GAPDH	98	OS	Kaplan–Meier	60	Indirectly	7
Du et al. [22]	2017	China	PCa	RT-PCR	GAPDH	62	OS	Kaplan–Meier	36	Indirectly	6
Xiao et al. [45]	2017	Ukraine	CRC	RT-PCR	GAPDH	70	OS	Multivariate	100	Indirectly	6
Wu et al. [16]	2017	China	ESCC	RT-PCR	GAPDH	64/63	OS	Multivariate	100	Indirectly	8
Chen et al. [17]	2017	China	CRC	RT-PCR	GAPDH	58/57	OS	Kaplan–Meier	150	Indirectly	8
Wei et al. [21]	2017	China	PC	RT-PCR	GAPDH/RNU6B	32/32	OS	Multivariate	30	Indirectly	6
Du et al. [38]	2017	China	Glioma	RT-PCR	GAPDH	35/34	OS	Multivariate	36	Indirectly	6
Hu et al. [23]	2017	China	BC	RT-PCR	GAPDH	32/20	OS	Kaplan–Meier	50	Indirectly	6
Kobayashi et al. [20]	2016	Japan	CSCC	RT-PCR	GAPDH	24/25	OS	Multivariate	160	Indirectly	6
Chen et al. [19]	2016	China	GC	RT-PCR	GAPDH	54/52	OS	Kaplan–Meier	120	Indirectly	8
Song et al. [14]	2016	China	NPC	RT-PCR	GAPDH	76/32	OS	Kaplan–Meier	120	Indirectly	8

Abbreviations: BC, bladder cancer; CRC, colorectal cancer; CSCC, cervical squamous cell carcinoma; ESCC, esophageal squamous cell carcinoma; GC, gastric cancer; NPC, nasopharyngeal carcinoma; GAPDH, glyceraldehyde-3-phosphate dehydrogenase; RNU6B, RNA, U6 small nuclear 6B; OS, overall survival; PC, pancreatic cancer; PCa, prostate cancer.

**Table 2:** Summary of the comparison for the *P*-values for the association between lncRNA XIST and clinicopathological characteristics

Author	Year	Country	Tumor type	Sample	Total number	Age	Gender	Tumor size	LNM	DM	Differentiation	Stage	Expression
Wu et al. [16]	2017	China	ESCC	Tissue	127	0.286	0.410	0.320	-	-	0.831	0.596	Up
Wei et al. [21]	2017	China	PC	Tissue	64	0.798	0.317	0.006	0.131	0.079	-	0.023	Up
Du et al. [22]	2017	China	PCa	Tissue	62	0.324	-	-	<0.01	<0.01	-	0.012	Down
Hu et al. [23]	2017	China	BC	Tissue	52	0.540	0.658	0.028	0.042	-	-	0.012	Up
Ma et al. [40]	2017	China	GC	Tissue	98	0.175	0.651	0.006	0.002	-	-	0.005	Up
Mo et al. [37]	2017	China	HCC	Tissue	88	0.119	0.754	0.002	-	-	-	-	Up
Du et al. [38]	2017	China	Glioma	Tissue	69	0.921	0.537	0.003	-	-	-	<0.001	Up
Xiong et al. [39]	2017	China	BC	Tissue	67	0.389	-	0.393	-	0.901	0.418	0.036	Up
Chen et al. [19]	2016	China	GC	Tissue	106	0.253	0.648	0.023	0.013	0.011	0.326	0.016	Up
Fang et al. [15]	2016	China	NSCLC	Tissue	53	0.951	0.062	0.003	0.511	-	0.0317	0.0002	Up
Kobayashi et al. [20]	2016	Japan	CSCC	Tissue	49	0.12	-	0.87	0.110	-	-	0.810	Down
Tantai et al. [46]	2015	China	NSCLC	Tissue	32	0.549	0.717	0.010	0.001	-	-	-	Up

Abbreviations: BC, bladder cancer; CSCC, cervical squamous cell carcinoma; DM, distant metastasis; ESCC, esophageal squamous cell carcinoma; GC, gastric cancer; HCC, hepatocellular carcinoma; LNM, lymph node metastasis; NSCLC, non-small-cell lung cancer; PC, pancreatic cancer; PCa, prostate cancer.

### Association between XIST and prognosis

**Figure 2:**
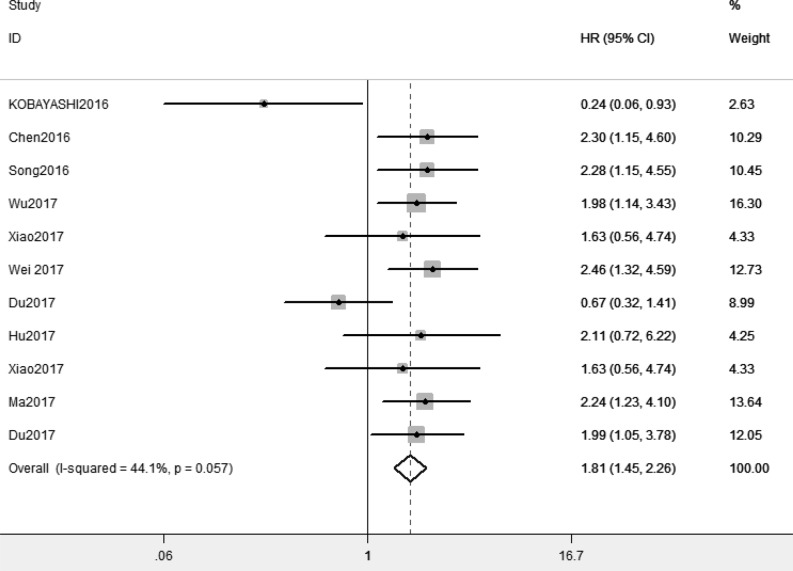
Forest plot for the relationship between XIST expression and overall survival

**Figure 3:**
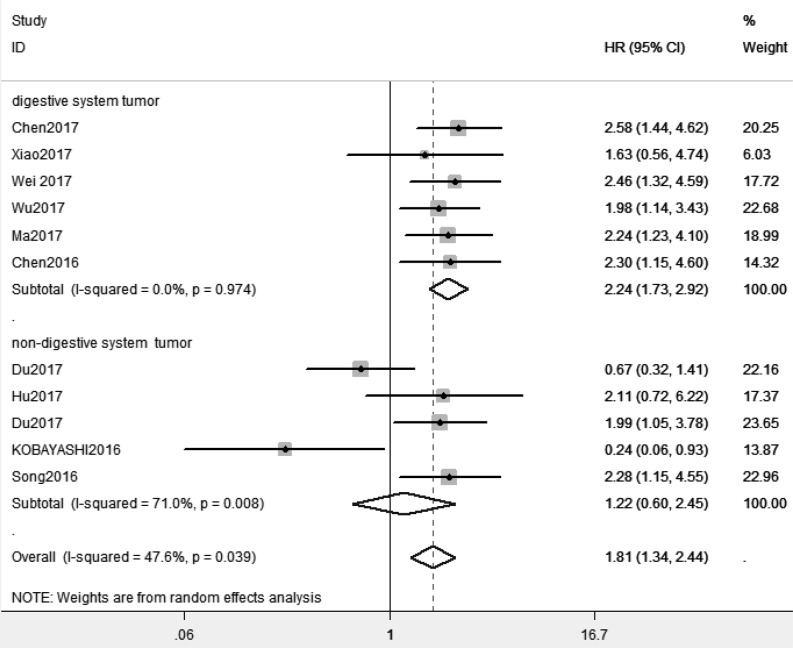
Subgroup analysis of overall survival by cancer types

**Figure 4:**
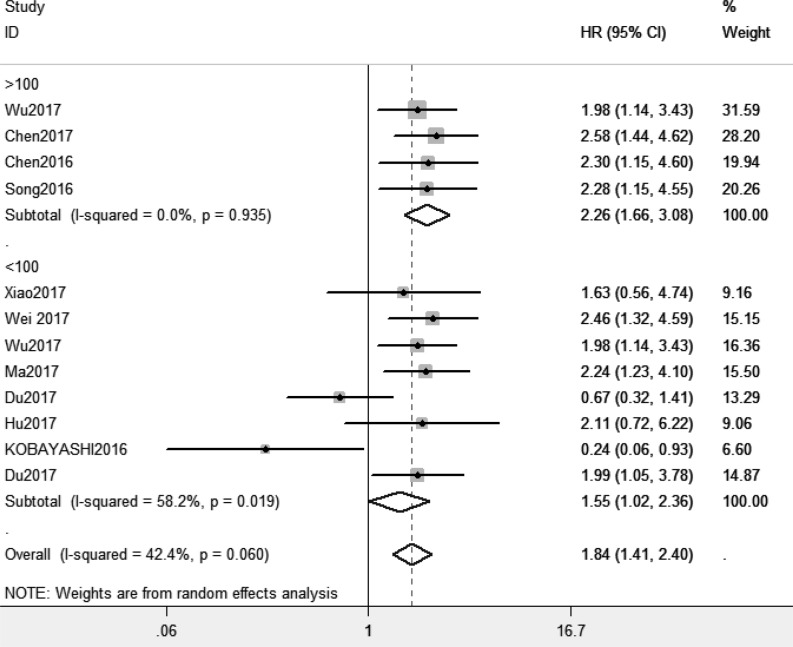
Subgroup analysis of overall survival by sample sizes

**Figure 5:**
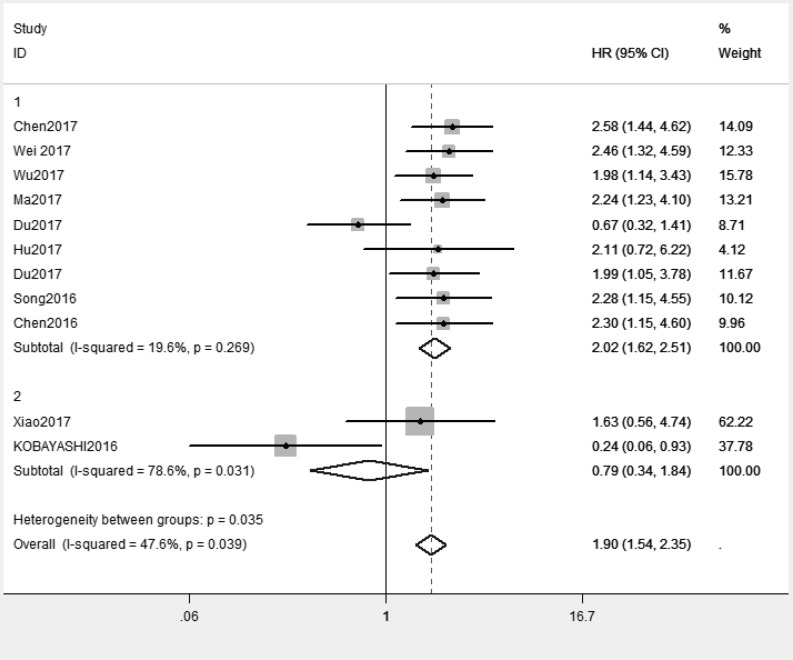
Subgroup analysis of overall survival by regions

Because of absence of obvious heterogeneity amongst those 11 studies (*I^2^* < 50%), the fixed-effect model was used to analyze the pooled HR and its 95% CI. The results showed that elevated XIST expression levels predicted poor prognosis (pooled HR = 1.81, 95% CI: 1.45–2.26) (Figure 2). Afterward, the subgroups were divided by cancer types, sample sizes and, regions (Figures 3–5). According to the subgroup analysis, XIST could predict worse prognosis in digestive system tumors (pooled HR = 2.24, 95% CI: 1.73–2.92) than in non-digestive system tumors (pooled HR = 1.22, 95% CI: 0.60–2.45). Further, we did not observe significant heterogeneity in digestive system tumors (*I^2^* = 0.0%, *P*=0.990), indicating that the results in digestive system tumors were consistent and convincing. We also observed that the studies with sample sizes in excess of 100 were less heterogeneous than those studies whose sample sizes were less than 100, which suggested that increasing the sample size could be helpful to reduce the resources of heterogeneity. Regarding subgroup analysis on the basis of regions, we came to know that the studies from China (*I^2^*= 19.6%, *P*=0.269) were less heterogeneous than those from Ukraine and Japan (*I^2^* = 78.6%, *P*=0.031). The results were significant in China (pooled HR = 2.00, 95% CI: 1.57–2.56), whereas no significant result was observed in Ukraine and Japan (pooled HR = 0.66, 95% CI: 0.10–4.29). The results of subgroup analysis were summarized in Table 3.

**Table 3:** Subgroup analysis of the pooled HRs with XIST expression in patients with cancer

Subgroup analysis	Studies (*n*)	Number of patients	HR (95% CI)	Heterogeneity		
				*I^2^* (%)	*P*	Model
**Cancer type**						
Digestive system tumor	6	580	2.24 (1.73, 2.92)	0.0%	0.974	Random effects
Non-digestive system tumor	5	340	1.22 (0.60, 2.45)	71%	0.008	Random effects
**Sample size**						
>100	4	552	2.26 (1.66, 3.08)	0.0%	0.935	Random effects
<100	7	368	1.55 (1.02, 2.36)	58.2%	0.019	Random effects
**Region**						
China	9	801	2.00 (1.57, 2.56)	19.6%	0.269	Random effects
Japan and Ukraine	2	119	0.66 (0.10, 4.29)	78.6%	0.031	Random effects

### Correlation of XIST and clinicopathological characteristics

Twelve studies were enrolled for clinicopathological characteristics consisting of 867 patients. Generally, age and gender had no effect on the expression levels of XIST (*P*>0.05). Additionally, the levels of XIST generally varied with tumor size (*P*<0.05). Studies with information of lymph node metastasis, distant metastasis, differentiation, and clinical stages were retrieved into OR analysis. We observed that high XIST expression was associated with positive distant metastasis (pooled OR = 2.28, 95% CI: 1.29–4.02) and clinical stages (pooled OR = 2.25, 95% CI: 1.67–3.03). There existed a significant heterogeneity for lymph node metastasis subgroup (*I^2^* = 75.3%, *P*=0.000) across studies included, therefore the random model was used to analyze the relationship between the expression levels of XIST and lymph node metastasis subgroup. And no significant results were observed (pooled OR = 1.86, 95% CI: 0.83–4.15). With respect to differentiation, we also observed no significance (pooled OR = 1.46, 95% CI: 0.92–2.33). The relationship between clinicopathological parameters and XIST expression levels was presented in Table 4. However, more studies were required to draw conclusive conclusions.

**Table 4:** Pooled ORs for the relationship between XIST expression levels and clinicopathological parameters

Category	Number of patients	OR (95% CI)	Heterogeneity		
			*I^2^* (%)	*P*	Model
Lymph node metastasis (yes compared with no)	516	1.86 (0.83, 4.15)	75.3%	0.000	Random model
					
Distant metastasis (yes compared with no)	299	2.28 (1.29, 4.02)	0.0%	0.532	Fixed model
					
Differentiation (poor compared with moderate/well)	353	1.46 (0.92, 2.33)	16.8%	0.308	Fixed model
					
Tumor stage (III/IV compared with I/II)	747	2.25 (1.67, 3.03)	61.7%	0.023	Random model
					

### Sensitivity analysis and publication bias

**Figure 6:**
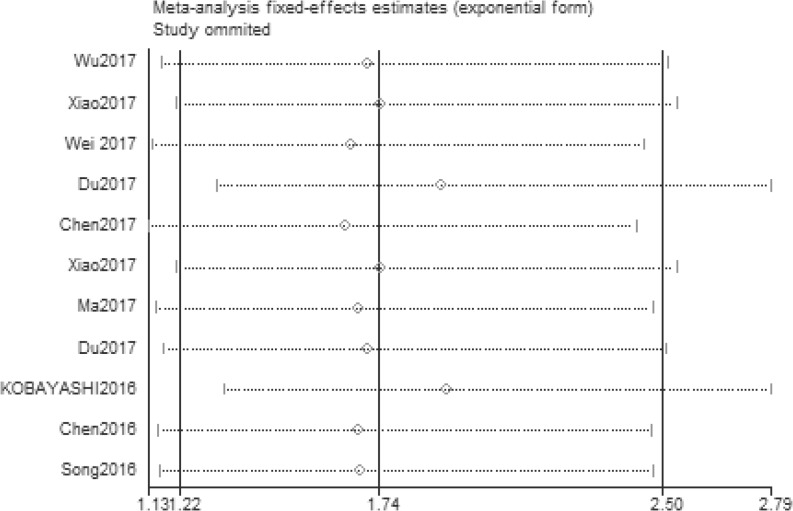
Sensitivity of XIST expression for overall survival

**Figure 7:**
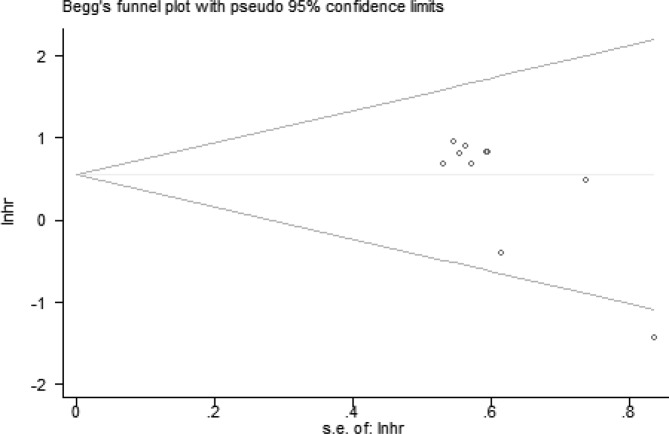
Funnel plot of the publication bias for overall survival

The stability and reliability of the results were evaluated by sensitivity analysis. The results suggested that the conclusions were stable and reliable because the pooled HR was not significantly affected by any individual study (Figure 6). And we evaluated the potential publication bias with Begg’s test (*P*=0.024). *P*<0.05 indicated that there existed publication bias (Figure 7). Only English papers and positive studies might generate publication bias.

### Reliability and conclusiveness of composite results

**Figure 8:**
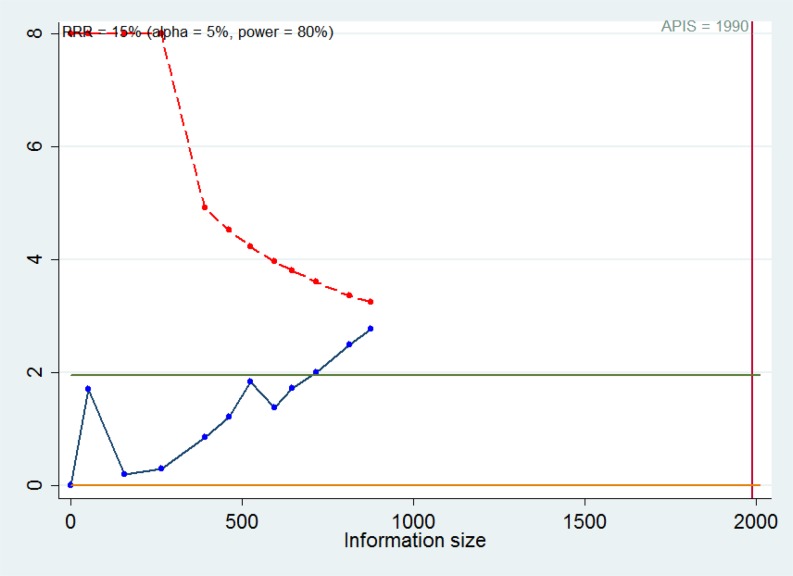
TSA for overall survival based on APIS

TSA was performed to estimate the sample size needed (Figure 8). We assumed a 15% RRR with 80% power and two-sided α values of 0.05 to determine APIS for overall survival. The cumulative Z-curve (the blue one) crossed the traditional Z-curve (Z = 1.96), but failed to cross the TSA monitoring boundary (the red one), suggesting that the more trials were needed to confirm the positive result. For our meta-analysis, more well-designed studies with large sample sizes are necessary to improve the reliability and conclusiveness of composite results.

## Discussion

It is discovered that only 2% of the genomic sequences can code proteins, most of which are transcribed into non-coding RNA once considered to be the ‘junk RNA’ without biological function [25]. The development of high-throughput RNA sequencing technology provides an opportunity for us to discover non-coding RNA genes in great numbers [26]. Via interaction with DNA, RNA, or proteins, lncRNAs produce an effect on proliferation [27], potential invasion [28], resistance to radiation and drugs [29], and reprogrammed energy metabolism [30,31]. Recently, accumulating evidence indicates that lncRNAs were closely associated with many diseases, such as cardiovascular diseases [32], autoimmune diseases [33], diabetes mellitus [34], and so on. Meanwhile, an increasing number of cancer-related lncRNAs are gradually characterized. Due to high sensitivity, specificity, and convenient detection in the body fluids, lncRNAs have great potential to be promising biomarkers for early detection and accurate prognosis for cancer patients [35].

The mechanism underlying the relationship between XIST and cancer outcome is uncertain. XIST is involved in X-chromosome inactivation in the cells of females and allows X-chromosome equilibration in males and females [36]. Loss of X-chromosome inactivation and abnormal expression of XIST are commonly observed in various cancers. The ceRNA regulatory network, in which XIST can compete for endogenous miRNAs was confirmed in nasopharyngeal carcinoma, esophageal squamous cell carcinoma, gastric cancer, pancreatic cancer, prostate cancer, hepatocellular carcinoma, glioma, bladder cancer [14,19,21–23,37–40]. XIST could function as endogenous miRNA sponges to combine different miRNAs in different types of cancers, thus regulating proliferation, progression, and metastasis of tumors. In addition, Fang et al. [15] found that XIST could mediate its oncogenic effects through epigenetically silencing the expression of Kruppel-like factor (KLF2) via directly binding with enhancer of zeste homolog 2 (EZH2) in non-small-cell lung cancer. Furthermore, epithelial–mesenchymal transition (EMT) is a crucial step in tumor progression and metastasis [41]. XIST could mediate EMT by up-regulating epithelial markers (E-cadherin and β-catenin) and down-regulating mesenchymal markers in colorectal cancer [42]. XIST was involved in pathways related to tumorigenesis and cancer development, thus it had great therapeutic value and might contribute to personalized medicine therapy. One of the main strategies was using siRNAs to inhibit its expression. But the method is facing numerous challenges in clinical currently [43].

In order to combine the results of former studies about XIST and cancers, we elucidated the relationship between XIST expression levels and prognosis and also clinicopathological characteristics in cancer patients in the present meta-analysis. First, we performed to investigate the prognostic value of XIST in all kinds of tumors. The analysis showed the pooled HR was 1.81 (95% CI: 1.45–2.26), which suggested that elevated XIST expression levels were potentially related to poor prognosis. Then we observed higher predictive value of XIST in digestive system tumors (pooled HR = 2.24, 95% CI: 1.73–2.92) than in non-digestive system tumors (pooled HR = 1.22, 95% CI: 0.60–2.45) by subgroup analysis. Our meta-analysis showed elevated XIST expression levels in digestive system tumors predicted worse prognosis than in non-digestive system tumors for the first time. With regard to clinicopathological characteristics, our results indicated that elevated expression levels of XIST represented a greater possibility of distant metastasis (pooled OR = 2.28, 95% CI: 1.29–4.02) and high tumor stage (pooled OR = 2.25, 95% CI: 1.67–3.03). However, further large-scale studies should be conducted.

We cannot neglect that there exists some limitations in our analysis. First, more well-designed studies with large sample sizes need to be included in analysis. Second, some of the HRs were calculated by reconstructing survival curves rather than directly obtaining from the primary data. Third, most enrolled studies have positive results, but those with negative results were generally less likely to be published. In addition, patients included in this meta-analysis mostly came from People’s Republic of China, which might diminish the overall effect of the results. Finally, our studies were only English papers, which might generate publication bias. Despite the limitations mentioned above, our meta-analysis is the first to reveal that lncRNA XIST has more predictive value in digestive system tumors, which suggest that lncRNA XIST can become a potential prognostic marker and predict cancer outcome in digestive system tumors. Well-designed studies related to specific cancer types with large sample sizes are required to confirm the prognostic value of lncRNA XIST in digestive system tumors. The cancer prevalence is inevitably increasing worldwide. Surveillance and monitoring systems should be strengthened to reverse the global trend. To fight against cancers, it must be a top priority to improve surveillance and monitoring levels [44]. Thus, the present study is clinically significant in exploring reliable biomarkers which can predict prognosis in malignant cancers.

## Abbreviations

APIS: a *priori* information size
EMT: epithelial–mesenchymal transition
HR: hazard ratio
lncRNA: long non-coding RNA
OR: odds ratio
RRR: relative risk reduction
TSA: trial sequential analysis
XIST: X-inactive specific transcript
95% CI: 95% confidence interval
